# Supplementary figures and images for: Randomly connected networks generate emergent selectivity and predict decoding properties of large populations of neurons
Source: PLoS Comput Biol. 2020 May 7;16(5):e1007875. doi: 10.1371/journal.pcbi.1007875 (PMC7237045; doi:10.1371/journal.pcbi.1007875)

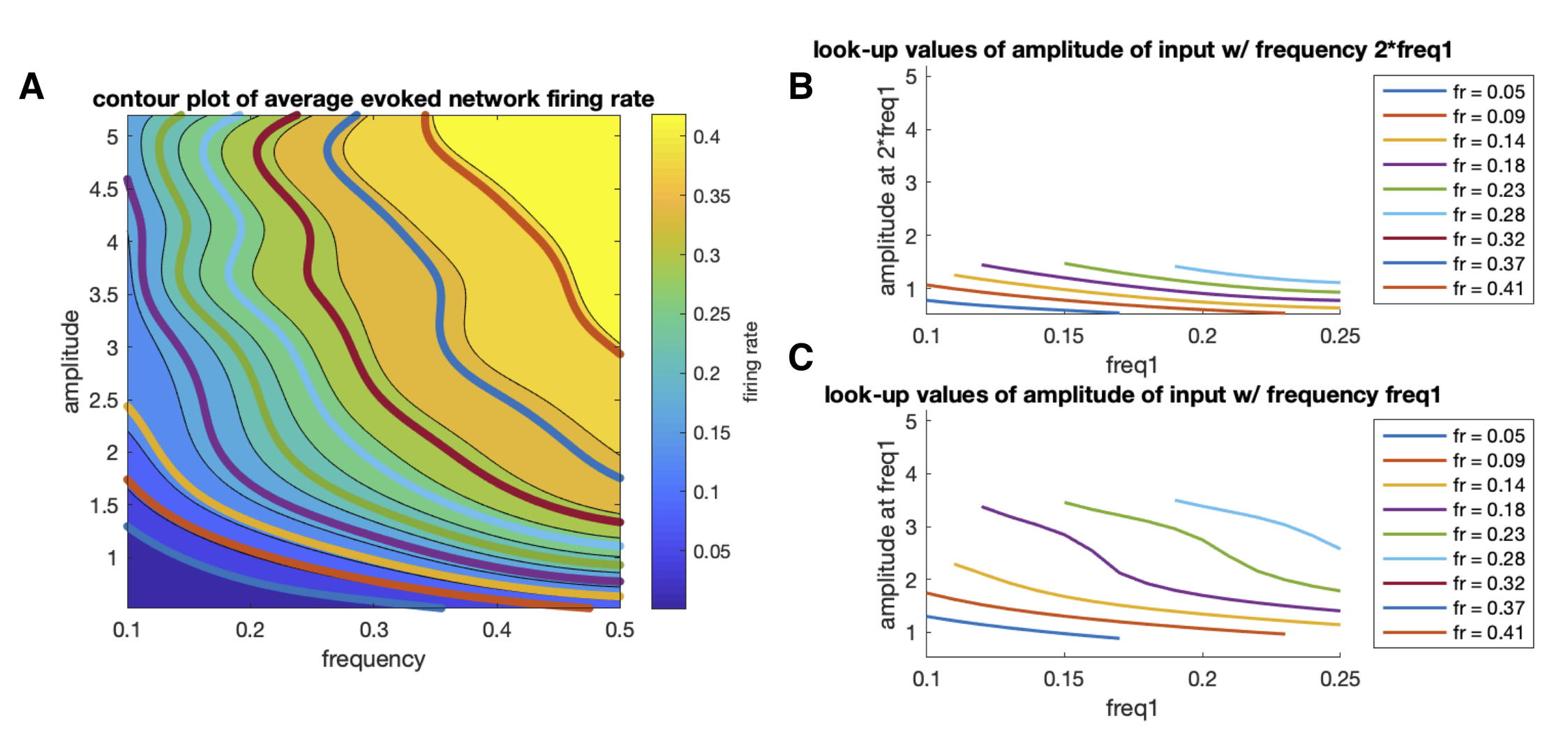

Supplement: S1 Fig — A: Surface is a spline interpolation of average (over time and neurons) firing rate across 25 combinations of amplitude and frequency. Color on parula scale indicates firing rate (blue to yellow). Lines show contours at fixed average firing rate. For each firing rate (indicated by line color), we extract amplitudes and frequencies on the corresponding contour. B: Amplitudes for the high-frequency (freq2 = 2*freq1) input, plotted against the lower frequency (freq1). C: Amplitudes for the low-frequency input, plotted against the lower frequency (freq1). The highest firing rate contour that is defined over freq1 = 0.14 to 0.2 is simulated. Units of frequency are per τ; multiplication by 50 converts to Hz. (TIF) [file pcbi.1007875.s001.tif]

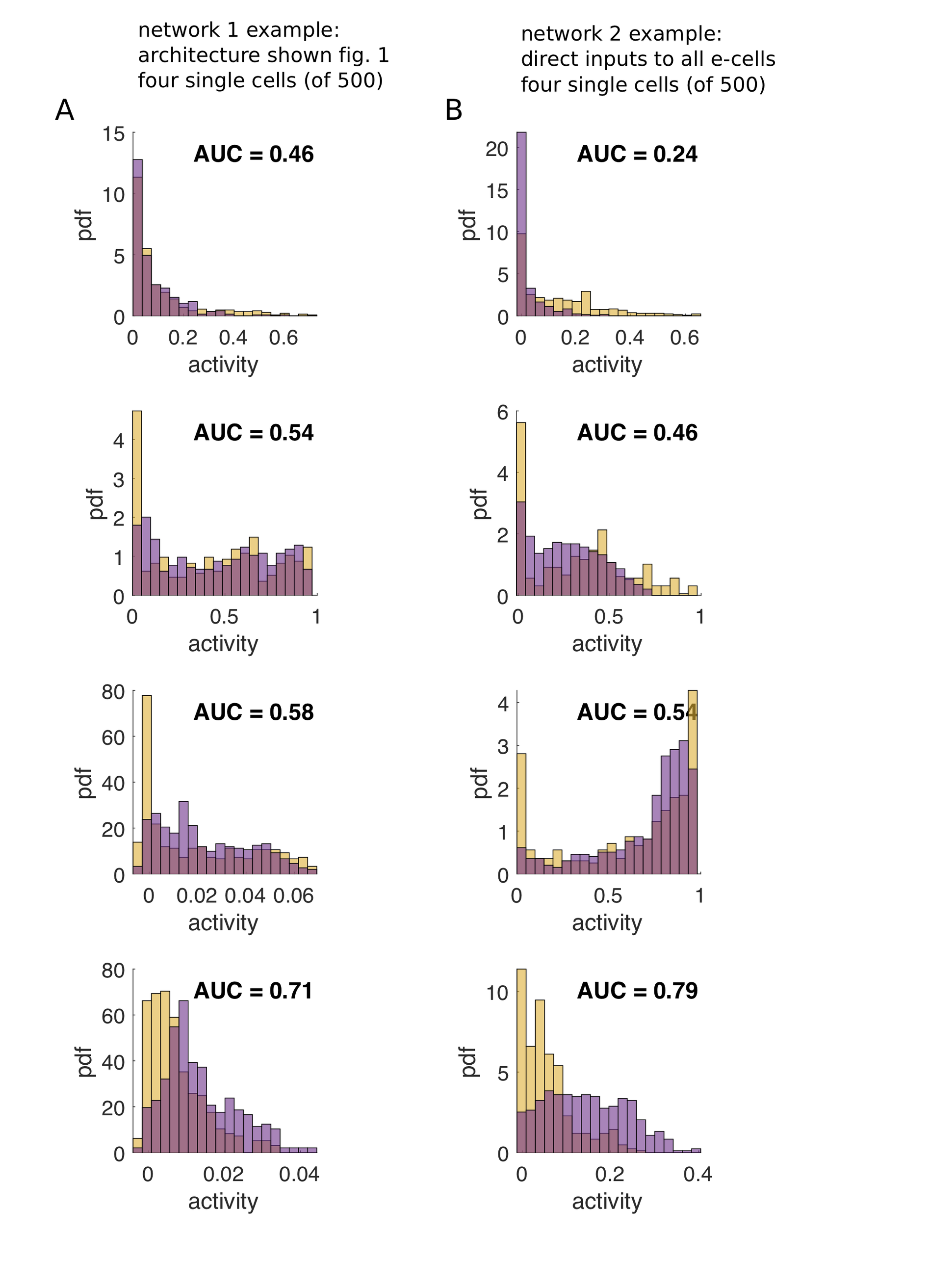

Supplement: S2 Fig — Example histograms of single-cell firing rate for the low- (yellow) and high-frequency (purple) inputs, with the area under the receiver-operator curve (AUC) statistic indicated. AUC values close to 0.5 are non-selective. A: (column) examples from one of the networks examined in Figs 1 to 3. B: (column) examples from a network receiving inputs to all excitatory cells (Fig 4). (TIF) [file pcbi.1007875.s002.tif]

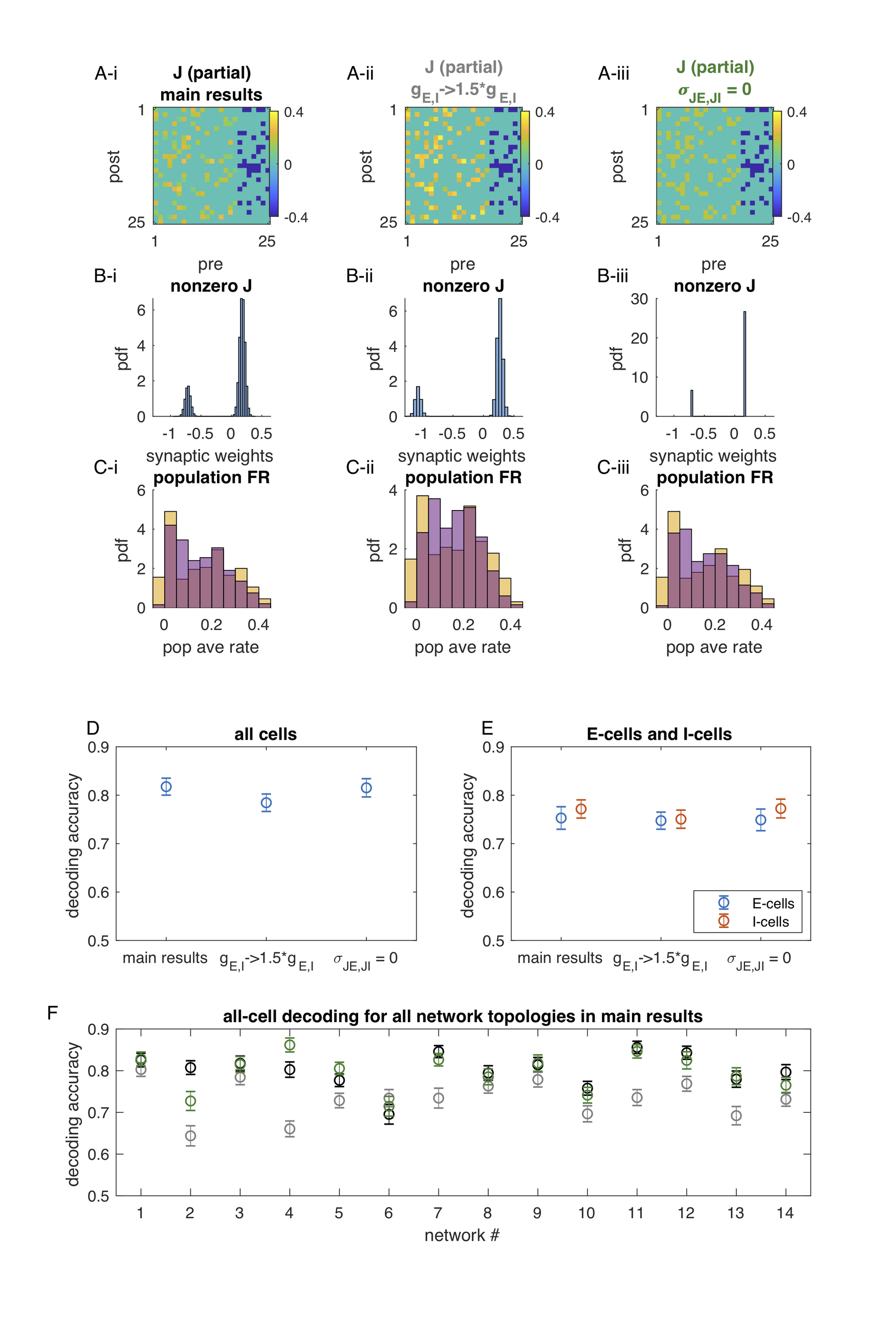

Supplement: S3 Fig — Fixing network topology (i.e., which elements of J are non-zero), we simulated three networks: with original weights (i), with all synaptic weights scaled by a factor of 1.5, (ii) and with homogeneous excitatory and homogeneous inhibitory synaptic weights (iii). A: Image of network connectivity for 25 (of 500 total) neurons showing that the topology was kept the same for each simulation. B: Distribution of non-zero excitatory and inhibitory weights in the network. Note that there are approximately four times as many excitatory weights, but they are on average a quarter of the strength of inhibitory weights. C: Histogram of stimulus 1 (yellow) and stimulus 2 (purple) population firing rates for each parameter scaling. Firing rates are matched for each simulation individually; these are operating over a similar population firing rate range. D: Decoding accuracy of the full population in each network. E: Decoding accuracy of excitatory and inhibitory cells in each network. D and E show that decoding accuracy persists after a drastic parameter change, for this network topology. F: Decoding performance for all simulated networks. (TIF) [file pcbi.1007875.s003.tif]

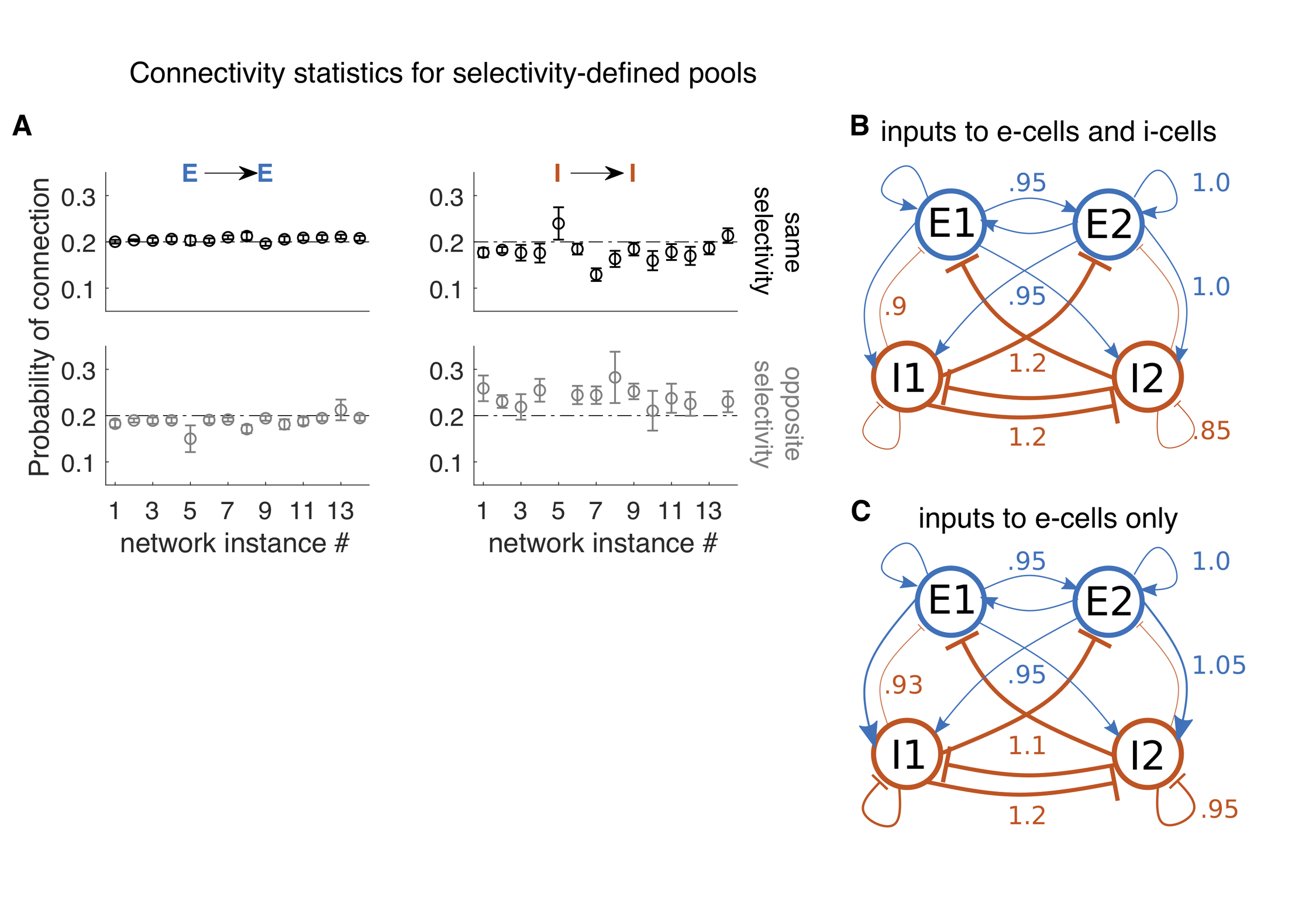

Supplement: S4 Fig — A: Average probability of connection between pairs of cells with the same selectivity (top row) and with opposite selectivity (bottom row) for pairs of excitatory cells (left column) and pairs of inhibitory cells (right column). B: Summary diagram of connectivity for the network when cells are divided into pools based on selectivity. Blue arrows: excitatory connections. Red end-stop lines: inhibitory connections. Weights label the probability of connection relative to the baseline (random chance) probability (1.0). Standard error across network simulations is 0.05 for inhibitory connections and 0.02 for excitatory ones. Connection probabilities are averaged across all networks in which there were cells in each of the four pools (excluding network instance 13, see A, lower right) and symmetrized across selectivity pools (i.e., the probability of connection from E1 to E2 is the same as the probability from E2 to E1, and from E1 to I1 is the same as from E2 to I2, and so on). Color of number (blue/red) matches the color of the connection drawn in the graph. C: Same diagram as in B, but for the network that only receives inputs to e-cells. Both cases show excess connectivity between pools I1 and I2 and from I1 to E2 (and from I2 to E1). (TIF) [file pcbi.1007875.s004.tif]

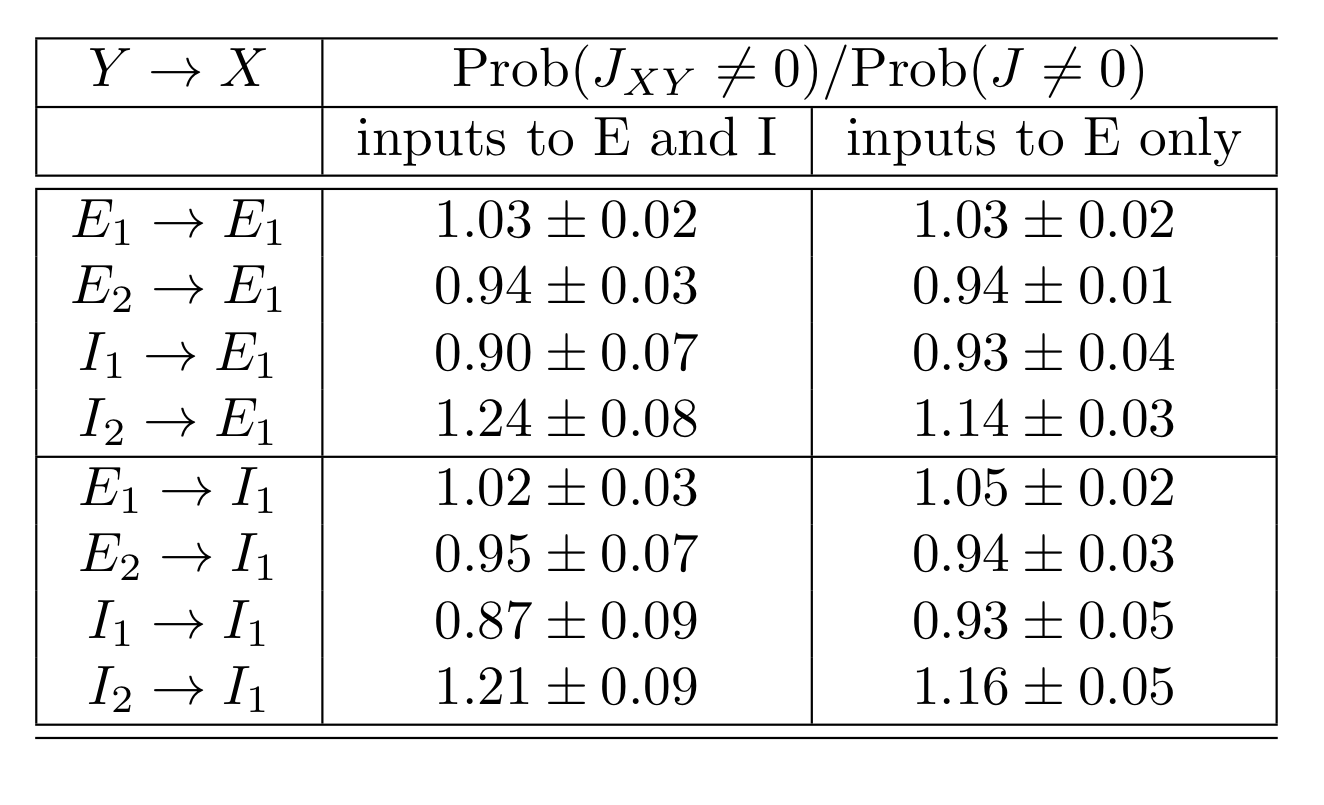

Supplement: S1 Table — For each network, pairs of neurons were randomly connected with probability p = 0.2. Following simulation, cells are divided into pools based on cell “type” (e-cells or i-cells) and selectivity (stimulus 1 and 2), yielding four pools: E1, E2, I1, and I2. The average probability of connection between each of the pools was calculated. Table entries are averages ± standard error across networks, normalized by the baseline probability of connection (0.2). Entries larger than 1 indicate more connections than the baseline expectation of 0.2. (TIF) [file pcbi.1007875.s005.tif]
